# Elevated frequency of abnormalities in barn swallows from Chernobyl

**DOI:** 10.1098/rsbl.2007.0136

**Published:** 2007-04-17

**Authors:** A.P Møller, T.A Mousseau, F de Lope, N Saino

**Affiliations:** 1 Laboratoire de Parasitologie Evolutive, CNRS UMR 7103, Université Pierre et Marie CurieBât. A, 7ème étage, 7 quai Street Bernard, Case 237, 75252 Paris Cedex 05, France; 2 Department of Biological Sciences, University of South CarolinaColumbia, SC 29208, USA; 3 Departamento de Biología Animal, Facultad de Ciencias, Universidad de ExtremaduraAvda. Elvas s/n, 06071 Badajoz, Spain; 4 Dipartimento di Biologia, Sez. Zoologia Scienze Naturali, Università degli Studi di Milanovia Celoria 26, 20133 Milano, Italy

**Keywords:** *Hirundo rustica*, mutation, radiation, viability

## Abstract

Ever since the Chernobyl accident in 1986, that contaminated vast areas in surrounding countries with radiation, abnormalities and birth defects have been reported in human populations. Recently, several studies suggested that the elevated frequency of such abnormalities can be attributed to poverty and stress in affected human populations. Here, we present long-term results for a free-living population of barn swallows, *Hirundo rustica*, demonstrating the presence of 11 morphological abnormalities in populations around Chernobyl, but much less frequently in an uncontaminated Ukrainian control population and three more distant control populations. The presence of these abnormalities in barn swallows is associated with reduced viability. These findings demonstrate a link between morphological abnormalities and radiation in an animal population that cannot be attributed to poverty and stress. The most parsimonious hypothesis for abnormalities in animal and human populations alike is that the effects are caused by the same underlying cause, viz. radiation derived from the Chernobyl accident.

## 1. Introduction

Free-living organisms with morphological abnormalities have a frequency of less than 1% (Campbell & Lack 1985), because natural selection removes individuals with aberrant phenotypes. Hence, we should expect low frequencies of such aberrations in natural populations, even when environmental perturbations increase the frequency of abnormalities or birth defects. Human populations exposed to radiation from the Chernobyl nuclear power plant have elevated frequencies of abnormalities and birth defects (Feshchenko *et al*. 2002; UNDP/UNICEP 2002; Lazjuk *et al*. 2003; Scherb & Weigelt 2003; Chernobyl Forum 2005*a*,*b*). However, recent studies raised the possibility that such abnormalities may arise from deteriorating living conditions in general and not necessarily be due to radiation (Chernobyl Forum 2005*a*,*b*). In addition, worries about the effects of exposure to radiation among humans have been suggested to result in elevated levels of stress that potentially could cause an increase in the frequency of birth defects (Chernobyl Forum 2005*b*; Rosenthal 2005; Stephan 2005). In contrast, estimates of such abnormalities in animal populations exposed to radiation from Chernobyl cannot be said to suffer from these problems, and similarities in patterns of abnormalities in animal and human populations thus comprise a test of the effect of stressful conditions on abnormalities. Therefore, studies of abnormal phenotypes among animals can be used as a way to test for effects of radiation on the frequency of abnormalities.

Here, we present results from long-term studies of barn swallows in Chernobyl and control areas in Ukraine and in three other populations in Europe. With over 7700 individuals examined for external abnormalities, this provides the most extensive dataset on abnormalities in animals ever recorded.

## 2. Material and methods

We captured adult barn swallows in a contaminated area (Chernobyl (51°24′ N, 30°07′ E), open farmland within the exclusion zone or within a distance of 1 km from the border of the zone) and a control area (Kanev (49°06′ N, 33°33′ E), open farmland habitat similar to but approx. 220 km southeast of the Chernobyl area) in Ukraine during 1991, 1996, 2000 and 2002–2006. Additional control areas include Spain 2002–2006, Italy 2001–2006 and Denmark 1984–2006 (see Møller *et al*. (2005*a*, 2006*a*) for details about procedures).

Upon capture, all individuals from Ukraine and Denmark were examined by APM, from Spain by FdL and Italy by NS, for the presence of deviations from normal phenotype by careful examination while recording 20 morphological measurements and three measures of abundance of parasites on each individual (Møller *et al*. 2006*a*). The total procedure lasted 5–6 min per bird, and procedures were similar in all study sites because APM had instructed FdL and NS. Similarity in procedure was reflected by a high degree of repeatability of measurements among measurers (Møller *et al*. 2006*a*). Furthermore, APM found a similar frequency of partial albinos in Denmark during 1989–1990 and 1991–1992 (*Χ*_1_^2^=0.04, *p*=0.84), suggesting a similar search effort for albinistic individuals before and after initiation of the study in Chernobyl. Nestlings were inspected for abnormalities during 1996, 2000 and 2002–2006, restricted to individuals that were at least 10 days old to allow a check of the plumage for the presence of deviating coloration.

We identified the following 11 abnormalities that deviate from common morphology, based on our extensive experience from handling more than 20 000 barn swallows (figure 1): (i) Partial albinism refers to the presence of single or a few white feathers in the otherwise reddish brown or dark blue plumage (data for 1991, 1996 and 2000 previously reported by Møller & Mousseau 2001). This trait is maintained across moult in adults (Møller & Mousseau 2001). (ii) Aberrant coloration of plumage refers to a mottled plumage of the head (Møller & Mousseau 2001). (iii) Red coloration of chest refers to the presence of reddish brown coloration below the blue breast stripe. (iv) Blue coloration in the red face refers to blue spots in the normally reddish brown coloration of the head. (v) Deformed toes refer to crippled or malformed toes. (vi) Deformed beak refers to beaks that cannot be closed due to bent edges or malformed beaks. (vii) Tail feathers with non-fused barbs refer to tips of the outermost tail feathers with brushy appearance due to the absence of functional barbs. (viii) Bent tail feathers refer to lyre-shaped outermost tail feathers. (ix) Tumours were externally clearly visible lumps of hard tissue (with a diameter of more than 1 cm) on the outside of the body. (x) Deformed air sacks were observed in a female barn swallow with a completely swollen breast. (xi) Deformed eyes were partly covered by swollen or irregularly shaped eyelids. No individual had more than one abnormality.

We tested for deviations from a random distribution of abnormalities, using log-likelihood ratio tests (SAS 2000). Likewise, we used log-likelihood ratio tests to compare the frequency of abnormalities in nestlings and adults, and the number of survivors among individuals with and without abnormalities. We used logistic regression to test for temporal trends in abnormalities (SAS 2000).

## 3. Results

The 11 different categories of external malformations in barn swallows from Chernobyl ranged in frequency from 0.09% malformed air sacks to 13.69% partially albinistic plumage (table 1 in the electronic supplementary material). Only partial albinism, deformed toes and tumours were also found in control populations, albeit at a much lower frequency than in Chernobyl. If abnormalities were randomly distributed among contaminated and control populations, we should expect the number of abnormal individuals to reflect the number of individuals examined in each population. However, there were many more conditions recorded in Chernobyl than in the four control areas despite the opposite being expected, suggesting that abnormalities were non-randomly distributed among populations. Likewise, the frequency of individuals with abnormalities was much greater in Chernobyl than expected by chance (log-likelihood ratio test, *Χ*_1_^2^=156.86, *p*<0.0001), and that was the case for the frequency of both partial albinism (*Χ*_1_^2^=78.58, *p*<0.0001) and other abnormalities (*Χ*_1_^2^=140.39, *p*<0.0001).

If deteriorating conditions in Ukrainian agriculture were the cause of an elevated frequency of abnormalities in humans and animals alike, we would expect an increase in the frequency after 1990 associated with the breakdown of collective farming. However, the frequency of abnormalities among adult barn swallows from Chernobyl decreased significantly from 1991 to 2006 both for partial albinism and other abnormalities (figure 2; partial albinism: *Χ*_1_^2^=5.45, *p*=0.020; other abnormalities: *Χ*_1_^2^=5.62, *p*=0.018).

We should expect survival prospects of individuals with and without abnormalities in Chernobyl to be similar, if abnormalities were benign. We found evidence of natural selection against abnormalities in both nestlings and adults. First, a total of 33.5% of 248 nestlings from Chernobyl had abnormalities, while only 17.8% of 841 adults had any abnormalities (*Χ*_1_^2^=24.65, *p*<0.0001). Second, survival prospects for adults with and without abnormalities were estimated based on recaptures in subsequent years in the same sites. With capture probabilities above 90% and site philopatry of more than 99.9% (*N*>10 000 adults from Ukraine, Spain, Italy and Denmark; Møller *et al*. 2006*a*), we determined whether the probability of recapture in a subsequent year differed between individuals with and without abnormalities captured during 2002–2005. The survival probability of 48 birds with abnormalities from Chernobyl was estimated from recaptures. While the survival probability of 368 non-albino adults from Chernobyl was 0.279 (Møller *et al*. 2005*a*), the survival probability of birds with abnormalities was 0.063 (*Χ*_1_^2^=13.42, *p*=0.0002).

## 4. Discussion

This study showed an elevated diversity and frequency of abnormalities in barn swallows from Chernobyl compared with control populations in Ukraine and elsewhere. Furthermore, we found lower survival prospects for both nestlings and adults with abnormalities than for the average individual. Thus, the presence of abnormalities is associated with severe fitness costs. This is the first study showing that abnormalities in free-living animals are elevated in contaminated areas just as documented for humans (Feshchenko *et al*. 2002; Lazjuk *et al*. 2003; Scherb & Weigelt 2003). The cause of these effects is likely to be a combination of mutation rates having increased by up to more than a factor of 10 (Ellegren *et al*. 1997; Møller & Mousseau 2006) and elevated teratogenic effects of radioactive isotopes in the environment, possibly caused by depletion of antioxidants by radiation (Møller *et al*. 2005*b*; Møller & Mousseau 2006). We can exclude that the effects arose from non-radioactive pollution because all study areas were rural without any exposure to industrial pollution.

Radiation levels in the study areas in Chernobyl were one to two orders of magnitude above the background level (data reported in Møller *et al*. 2005*a*). Birds from Chernobyl are likely to have hatched at farms with elevated radiation levels, because mean natal dispersal distance is only 3 km per generation (Møller & Mousseau 2001). Likewise, birds from the Ukrainian control region are unlikely to have originated from the same areas as birds from the Chernobyl region, because the distance separating these two regions is 220 km, or more than 70 times the annual natal dispersal distance. If anything, the estimates of effects reported here are likely to be conservative, because there is more long distance dispersal into the Chernobyl area than the control area (Møller *et al*. 2006*b*).

We can think of no alternative explanations other than exposure to radiation that can have caused the observed patterns. There has been a general deterioration of farming in Ukraine since 1990. This should have negative effects in both control and contaminated areas, predicting increases in the frequency of abnormalities. We demonstrated a significant decrease, as expected from decreased levels of radiation during 1991–2006 due to natural decay of isotopes. Estimates of the frequency of abnormalities in birds could potentially have been overestimated if individuals with abnormalities are more likely to be caught. However, we can exclude the possibility of biased sampling because capture probability has been consistently high both in Ukraine and the non-Ukrainian control populations (Møller *et al*. 2005*a*, 2006*a*).

## Figures and Tables

**Figure 1:**
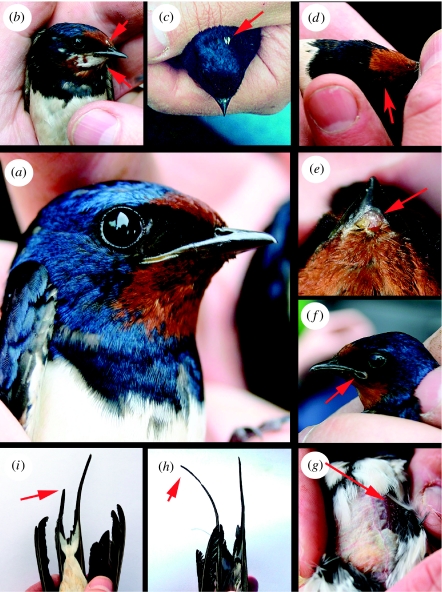
Photographs of abnormalities in barn swallows. (*a*) Normal phenotype. (*b*–*d*) Partially albinistic plumage. (*e*) and (*f*) Deformed beak. (*g*) Deformed air sacks. (*h*) and (*i*) Bent tail feathers.

**Figure 2:**
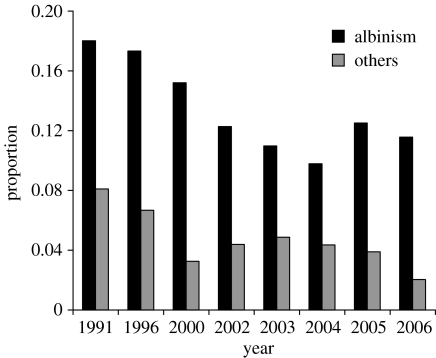
Frequency of partial albinism and other abnormalities in barn swallows in Chernobyl during 1991–2006.

## Supplementary Material

SupplMat.doc Table 1: Frequency (% (number)) of external morphological abnormalities in barn swallows from Chernobyl and four control populations
