# Supplementary material for: Elevated frequency of abnormalities in barn swallows from Chernobyl
Source: Biol Lett. 2007 Apr 17;3(4):414–7. doi: 10.1098/rsbl.2007.0136 (PMC1994720; doi:10.1098/rsbl.2007.0136)
Supplement: Supplementary file 1 [file rsbl20070136s17.doc]

Supplementary material:

Table 1. Frequency (% (number)) of external morphological abnormalities in barn swallows from Chernobyl and four European control populations.

| Condition | Chernobyl | Ukrainian control area | Denmark | Spain | Italy |
| --- | --- | --- | --- | --- | --- |
| Partial albinism | 13.32  (112) | 3.75  (20) | 4.87  (204) | 1.96  (11) | 4.06 (65) |
| Aberrant coloration of plumage | 0.28  (3) | 0 | 0 | 0 | 0 |
| Red coloration on chest | 0.28  (3) | 0 | 0 | 0 | 0 |
| Blue coloration in red face | 0.19  (2) | 0 | 0 | 0 | 0 |
| Deformed toes | 0.76  (8) | 0 | 0 | 0.18  (1) | 0.06  (1) |
| Deformed beak | 0.38  (4) | 0 | 0 | 0 | 0 |
| Tail feathers with non-fused barbs | 0.57  (6) | 0 | 0 | 0 | 0 |
| Bent tail feathers | 0.19  (2) | 0 | 0 | 0 | 0 |
| Tumours | 0.66  (7) | 0 | 0 | 0 | 0.19  (3) |
| Deformed air-sacks | 0.09  (1) | 0 | 0 | 0 | 0 |
| Deformed eyes | 0.19  (2) | 0 | 0 | 0 | 0 |
| *N* | 841 | 534 | 4198 | 562 | 1601 |
